# Supplementary material for: Identification of Genetic Risk Factors of Severe COVID-19 Using Extensive Phenotypic Data: A Proof-of-Concept Study in a Cohort of Russian Patients
Source: Genes (Basel). 2022 Mar 17;13(3):534. doi: 10.3390/genes13030534 (PMC8949130; doi:10.3390/genes13030534)
Supplement: Supplementary file 1 [file genes-13-00534-s001.zip › COVID Exomes - SI.pdf]

Identification of genetic risk factors of severe COVID-19 using extensive phenotypic data: a proof-of-concept study in a cohort of Russian patients

Sergey G. Shcherbak[1,5], Anton I. Changalidi[1,2,3], Yury A. Barbitoff[1,2,4], Anna Yu. Anisenkova [1,5], Sergei V. Mosenko[1,5], Zakhar P. Asaulenko[5,6], Victoria V. Tsay[5,7], Dmitrii E. Polev[4,5], Roman Kalinin[4,5,7], Yuri A. Eismont[5,7] Andrey S. Glotov[,1,4], Evgeny Y. Garbuzov[5], Alexander N. Chernov,[5,8], Olga A. Klitsenko[1,5,6], Mikhail O. Ushakov[4], Anton E. Shikov[5], Stanislav P. Urazov[5], Vladislav S. Baranov[4], Oleg S. Glotov[4,5,7]

1 - St. Petersburg State University, St. Petersburg, Russia
2 - Bioinformatics Institute, St. Petersburg, Russia
3 - ITMO University, St. Petersburg, Russia
4 - D.O. Ott Research Institute of Obstetrics, Gynaecology, and Reproductology, St. Petersburg, Russia
5 - City Hospital No. 40, St. Petersburg, Russia
6 - North-Western State Medical University named after Ivan Ivanovich (I.I.) Mechnikov, St. Petersburg, Russia
7 - Children's Scientific and Clinical Center for Infectious Diseases of the Federal Medical and Biological Agency, St. Petersburg, Russia
8 - Institute of Experimental Medicine, St. Petersburg, Russia

Supplementary Figures

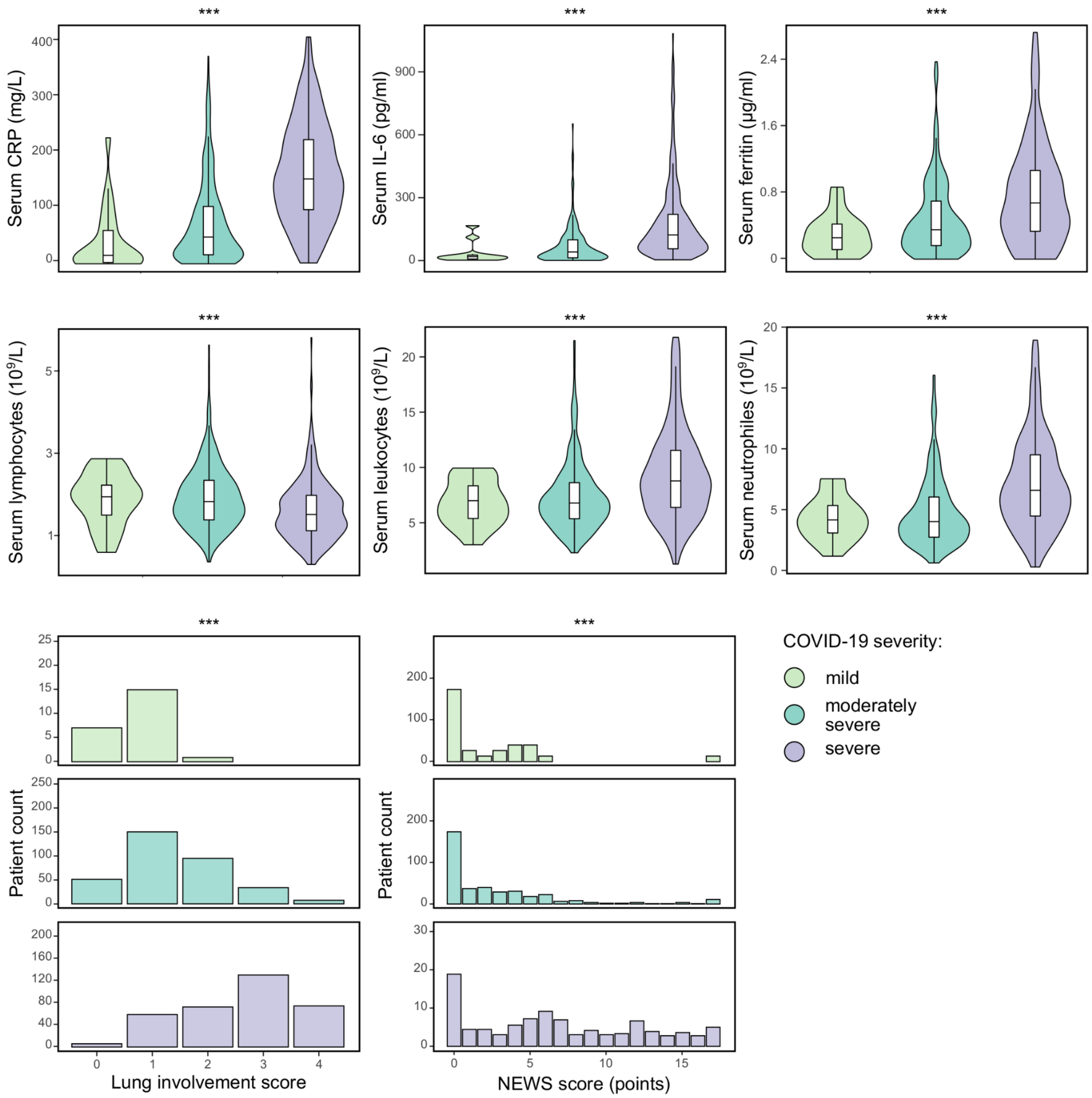

Figure S1. Distributions of selected quantitative traits for individuals with different degrees of disease severity in the cohort of 840 COVID-19 patients from Russia. Shown are the distributions of the serum C-reactive protein (CRP), interleukin-6, and D-dimer levels, CT-based lung involvement score (ranging from 0 to 4), counts of lymphocytes, leukocytes, and neutrophils in the blood samples, as well as the National Early Warning Score (NEWS). All values shown correspond to maximum values recorded during the course of hospitalization. Values exceeding three standard deviations from the population mean are omitted. *** - $p <$ 0.001 in Wilcoxon-Mann-Whitney test (for quantitative traits) or chi-squared test (for categorical traits).

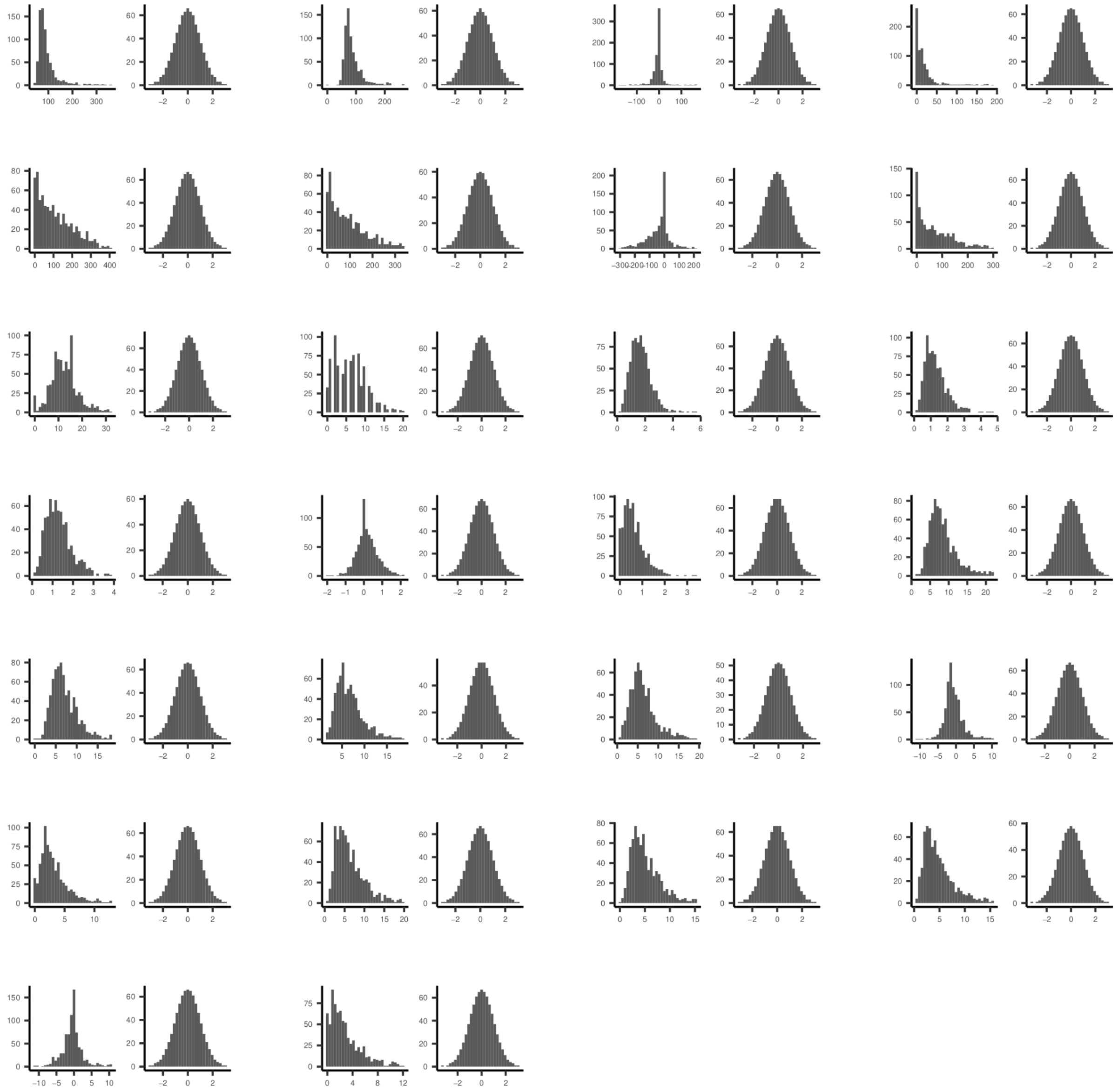

Figure S2. Distributions of the quantitative traits before and after IRNT transformation. Shown are histograms of the following traits (from left to right): top row - creatine levels (maximum; at admission; change (outcome vs. admission), differences (max. - min.)); second row - CRP levels (maximum; at admission; change (outcome vs. admission), differences (max. - min.)); third row - days at hospital, days from diagnosis to admission, lymphocyte count (maximum; at admission); fourth row - lymphocyte count (at third day from admission; change (outcome vs. admission; difference (max. vs. min.)), leukocyte count (maximum); fifth row - leukocyte count (at admission; at third day since admission; at seventh day since admission; change (outcome vs. admission)); sixth row - leukocyte count (difference (max. vs. min.)), neutrophil count (maximum; at admission; at first day; at third day); last row - neutrophil count (change (at outcome vs. at admission), difference (max. vs. min.).

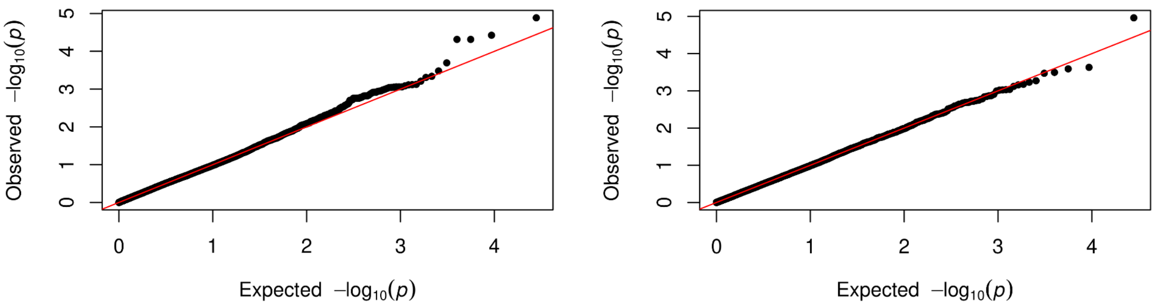

Figure S3. Quantile-quantile plots showing the results of the common variant association analysis for COVID-19 outcome (left), and severity (right).

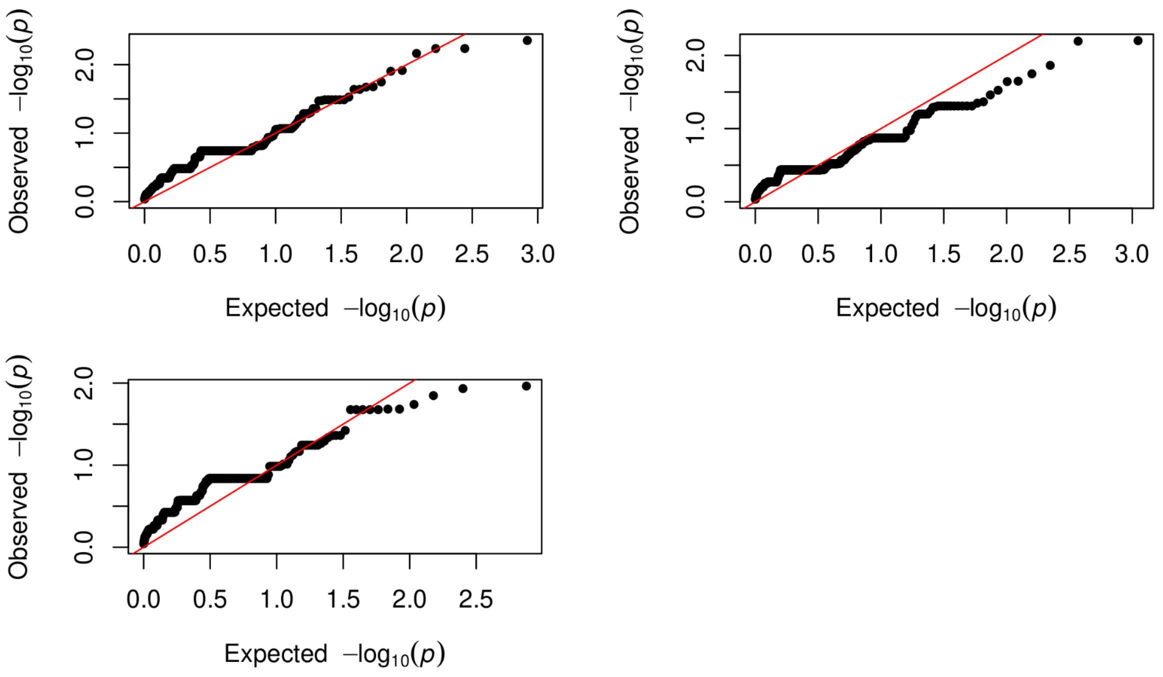

Figure S4. Quantile-quantile plots showing the results of the gene-level rare variant association analysis for COVID-19 outcome (top left), severity (top right), and cytokine storm (bottom left).). The comparison sample includes population controls from Barbitoff et al., 2019.
